# The irreplaceable role of medical massive open online courses in China during the COVID-19 pandemic

**DOI:** 10.1186/s12909-023-04315-z

**Published:** 2023-05-09

**Authors:** Hui Zhu, Jin Xu, Penghao Wang, Jia Bian, Zhijia Zhao, Hongyi Liu, Lindan Ji

**Affiliations:** 1 Department of Internal Medicine, Health Science Center, Ningbo University, Ningbo, Zhejiang 315211 People’s Republic of China; 2 School of Public Health, Health Science Center, Ningbo University, Ningbo, Zhejiang 315211 People’s Republic of China; 3 Academic Affairs Office, Ningbo University, Ningbo, Zhejiang 315211 People’s Republic of China; 4 Affiliated People’s Hospital of Ningbo University, Ningbo, Zhejiang 315211 People’s Republic of China; 5 Department of Biochemistry and Molecular Biology, School of Basic Medical Sciences, Health Science Center, Ningbo University, Ningbo, Zhejiang 315211 People’s Republic of China

**Keywords:** Massive open online course, Medical education, COVID-19

## Abstract

**Background:**

Massive open online courses (MOOCs) have become innovative open-learning approach in medical education. This study aimed to evaluate the dynamic changes in the construction and application of medical MOOCs before and after the coronavirus disease 2019 (COVID-19) pandemic in China.

**Methods:**

The dynamic changes of usages about medical MOOCs before and after 2020 were mainly searched on the Smart Education of China Higher Education platform, and the detailed learning profiles and outcome indicators were further analyzed using 40 national first-class medical MOOCs from ‘zhihuishu’ platform.

**Results:**

A total of 2,405 medical MOOCs were exported from the Smart Education of China Higher Education platform, of which 1,313 (54.6%) were launched since 2020. The total and average numbers of participants of 141 national first-class medical MOOCs peaked during the initial spread of COVID-19 in 2020. We further analyzed the dynamic usage of MOOCs from 2018 to 2022 based on 40 national first-class medical MOOCs launched on the ‘Zhihuishu’ platform. The findings revealed that the number of registered learners (3,240 versus 2,654), questions and answers (27,005 versus 5,116) and students taking the final examination (2,782 versus 1,995) per semester were significantly higher since 2020 compared to these before 2020. Especially, the number of registered learners, registered schools, questions and answers, and students participating in online discussion, taking the unit quiz, taking final examinations and passing final examinations all peaked in the 2020 spring–summer semester. Pearson's correlation analysis found that the number of questions and answers and the number of learners who participated in online discussion were both positively correlated with the number of students who passed the final examination, and the correlation was especially strong since 2020. Moreover, the number of publications on medical MOOC research has soared since 2020 and has maintained a continuous upward trend.

**Conclusions:**

High-quality medical MOOCs have been launched rapidly since the COVID-19 pandemic in China. The number of participants and online interactions of medical MOOCs peaked during the initial spread of COVID-19 in 2020. MOOCs are reliable and valid digital sources that facilitate medical higher education and play irreplaceable roles in emergency management.

**Supplementary Information:**

The online version contains supplementary material available at 10.1186/s12909-023-04315-z.

## Background

Massive open online courses (MOOCs) have become innovative open-learning landscapes [1, 2]. The distinctive features of MOOCs are their unlimited number of participants, free cost, online accessibility and full-course availability [3]. The first MOOC was offered by George Siemens and Stephen Downes in 2008. They conducted the open-online course ‘Connectivism and Connective Knowledge’ at the University of Manitoba and enrolled more than 2200 learners through various internet platforms [4, 5]. Early on, many educators argued that the conventional education system could no longer deliver extensive knowledge and information due to limited time, space, education infrastructure, teacher resources and curriculum development and evaluation [3]. Thus, MOOCs represented a revolutionary change in universal access to educational opportunities and have attracted wide attention from global institutes of higher education [6]. The new educational pattern spread rapidly to universities and colleges worldwide since 2012, and the New York Times called 2012 ‘the year of the MOOC’ due to the explosion in popularity of MOOCs [7].

In China, Tsinghua University launched the first MOOC platform, ‘Xuetang Online’, in October 2013 and offered the first batch of MOOCs [8]. Subsequently, the Online Education Research Center of the Ministry of Education (MOE) of China was officially established in 2014, and the number of online learners exceeded 180,000 in the same year [9]. Since then, MOOCs have expanded rapidly in China and have achieved wide implementation in various disciplines. In 2015, the MOE of China provided suggestions to strengthen the application and management of online open courses in colleges and universities, which further developed national high-quality MOOCs, optimized the public service platform for MOOCs and promoted the application of MOOCs [10]. To date, 1875 national first-class MOOCs have been accredited by the MOE, including 141 medical MOOCs [11]. China held a national MOOC conference and a global MOOC conference in 2019 and 2020, respectively, and formed Chinese models of MOOC development, including concepts, technologies, standards, methods and evaluations [12, 13]. Overall, the Chinese paradigm of MOOCs and online education is maturing. The Smart Education of China Higher Education platform (www.chinaooc.com.cn/) was formed on March 29, 2022. This national MOOC platform provides the largest and most comprehensive curriculum to college and social learners [14]. Recently, China facilitated more than 52,500 online MOOCs with approximately 370 million registered participants, and more than 330 million college students received MOOC credits [15]. In fact, China ranks first worldwide in terms of the number and application of MOOCs.

At the beginning of 2020, the coronavirus disease 2019 (COVID-19) pandemic spread worldwide and caused massive lockdowns because of the highly contagious nature of the virus. Subsequently, normal classroom-based teaching could not be maintained, and online teaching and learning became the major approach in higher education institutions [16]. In this situation, MOOCs were an excellent platform for high-quality online learning [17]. The MOE of China proposed that colleges and universities should implement online teaching as a crisis management response on February 5, 2020 [18]. This required teachers and learners to quickly explore and master the digital technologies and methods of MOOC platforms. The COVID-19 outbreak became a litmus test for MOOC efficiency in practice. In the era of the COVID-19 pandemic, China’s colleges and universities conducted unprecedented large-scale online teaching practice using MOOCs, which was a new "learning revolution" in higher education. The proportion of online and offline blended teaching modes in higher education in China increased from 34.8% before the COVID pandemic to 84.2% [19]. MOOCs proved to be strategic learning platforms for professional education rather than only complementary resources for formal education in colleges and universities.

Although MOOCs are currently popular in medical education and practice [20, 21], it remains controversial whether MOOCs have advantages over traditional teaching due to the complex curriculum [22]. Previously, MOOCs on medical topics did not have a universal teaching mode, and most medical MOOCs acted as complementary materials for formal courses in higher education [21]. This posed a challenge to the rapid implementation and integration of this new pedagogical approach in campus medical curricula during the COVID-19 pandemic [23, 24]. Our study aimed to evaluate the changes in the construction and application of medical MOOCs before and after the COVID-19 outbreak in 2020 and further predict the new trend of MOOCs in the medical education in the future.

## Methods

### Research design and data collection

**Fig. 1:**
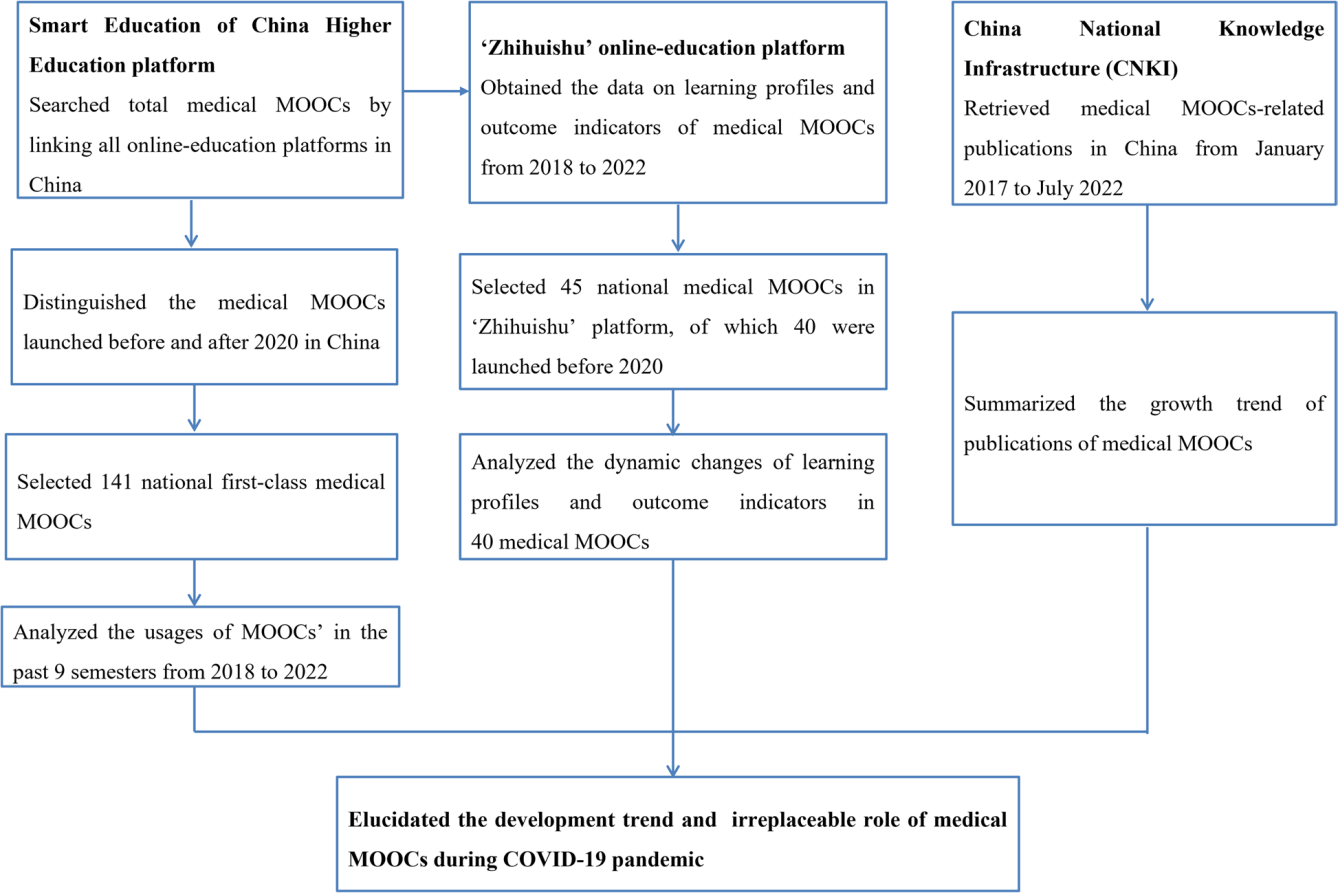
Flow chart for the overall process of research design and data retrieval

The Smart Education of China Higher Education platform (https://higher.smartedu.cn/) linked and integrated domestic online education platforms and gathered high-quality courses from colleges and universities in China. We searched the medical MOOCs from this platform and classified the medical MOOCs by the launch time which was before or after the COVID-19 pandemic in 2020. A total of 2,405 medical MOOCs were retrieved in the Smart Education of China Higher Education platform, of which 1092 were launched before 2020 and 1313 after 2020 (Fig. 1).

### The participants of medical MOOCs before and after 2020

The dynamic changes of participants were evaluated among 141 national first-class medical MOOCs from Smart Education of China Higher Education platform, and the total and average numbers of participants of medical MOOCs from the 2018 spring semester to the 2022 spring semester were extracted (Fig. 1).

### Outcome indicators of medical MOOCs learning

‘Zhihuishu’ was one of the top 3 MOOC platforms in China according to the total number of MOOCs and was linked with the Smart Education of China Higher Education platform. Importantly, ‘Zhihuishu’ is the only platform that provided detailed information about outcome indicators of medical MOOCs learning. The learning profiles and outcome indicators of medical MOOCs were further obtained from 45 national first-class medical MOOCs in ‘Zhihuishu’ (also called ‘Treenity’) online education platform (https://www.zhihuishu.com/), of which 5 were launched after 2020. Therefore, our subsequent analyses of the learning profiles and learning outcomes of medical MOOCs before and after the COVID-19 epidemic were performed using the data of 40 national first-class medical MOOCs launched before 2020 in ‘Zhihuishu’ platform. The main outcome indicators included the number of registered learners, registered schools, students participating in online discussion, questions and answers, and students taking the unit quiz, students taking the final examination, and students passing the final examination.

### Research publications on medical MOOCs in China

Publications, including academic journals, dissertations, conferences, newspapers, and books, on medical MOOCs in China from January 2017 to July 2022 were searched on China National Knowledge Infrastructure (CNKI) websites (https://www.cnki.net/) using the keywords ‘medical education’, ‘medical curriculum’, ‘medicine and health’, ‘traditional Chinese medicine’, ‘nursing’, ‘MOOCs’, ‘online learning’ and ‘online teaching’. The total number of MOOC-related publications in 2022 was estimated by the average number of published papers from January to July 2022.

Two reviewers independently retrieved and extracted all data, and the inconsistencies were collated and verified again by a third reviewer. The overall process of research design and data retrieval is summarized in the flow chart (Fig. 1).

All data are publicly available on the MOOCs platform. This study was exempt from the Ningbo University Medical Science Research Ethics Committee.

### Statistical analysis

The data on course application and evaluation are presented as the median and interquartile range (IQR), and the differences of the outcome indicators between these MOOCs before and after the COVID-19 pandemic were assessed with the Wilcoxon test. Pearson’s correlation analysis was conducted to identify the relationship between students who participated in online discussion as well as the records of questions/answers and students who passed the final examination. All statistical analyses were performed using SPSS 24.0 (SPSS Inc., Chicago, IL). All figures were plotted by GraphPad Prism 8.0 and the ggplot2 package for R software (version 4.2.0).

## Results

### Medical MOOCs rapidly launched after COVID-19 since 2020

**Fig. 2:**
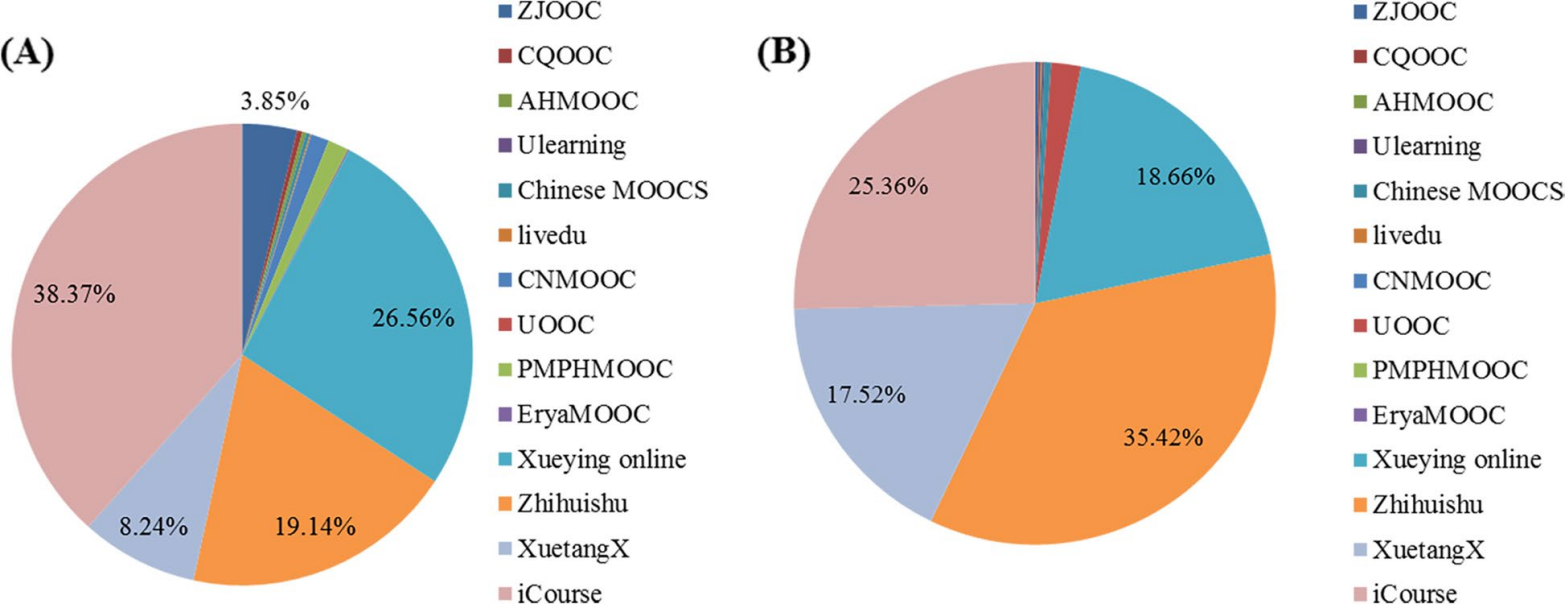
Medical MOOCs launched before and after 2020 on different online platforms. **A** MOOCs launched before 2020. **B** MOOCs launched in 2020

**Fig. 3:**
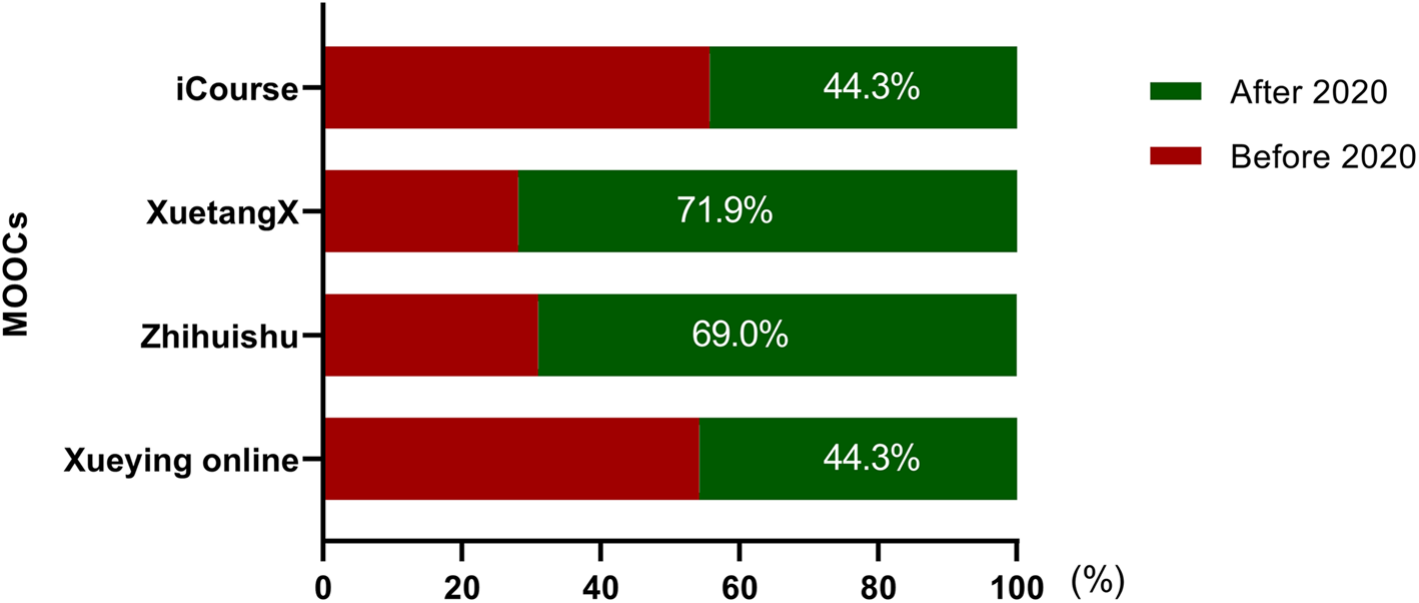
The proportions of medical MOOCs updated since 2020 in Zhihuishu, XuetangX, iCourse and Xueying Online. ‘Zhihuishu’ (https://www.xuetangx.com/), ‘XuetangX’ (https://www.xuetangx.com/), ‘iCourse’ (https://www.icourses.cn/home/) and ‘Xueying online’ (https://www.xueyinonline.com/) are the top four online platforms of medical MOOCs

A total of 2,405 medical MOOCs were exported from the Smart Education of China Higher Education platform, among which 1,092 MOOCs were launched before the COVID-19 epidemic and 1,313 MOOCs were launched in 2020. These MOOCs are available on 14 online education platforms of China and are primarily from ‘Zhihuishu’, ‘XuetangX’ (https://www.xuetangx.com/), ‘Xueying Online’ (https://www.xueyinonline.com/), and ‘iCourse’ (https://www.icourses.cn/home/) (Fig. 2). Among these platforms, the number of medical MOOCs on ‘Zhihuishu’ and ‘XuetangX’ were grown rapidly, and the proportion of those on ‘Zhihuishu’ and ‘XuetangX’ increased from 19.1% and 8.2% before the COVID-19 pandemic to 35.4% and 17.5% since 2020, respectively (Fig. 2). Moreover, approximately 70% of medical MOOCs on ‘Zhihuishu’ and ‘XuetangX’ were launched after 2020 (Fig. 3). These results indicated that the COVID-19 pandemic facilitated the development of MOOCs since 2020.

### The number of participants in medical MOOCs peaked in the 2020 spring–summer semester

**Fig. 4:**
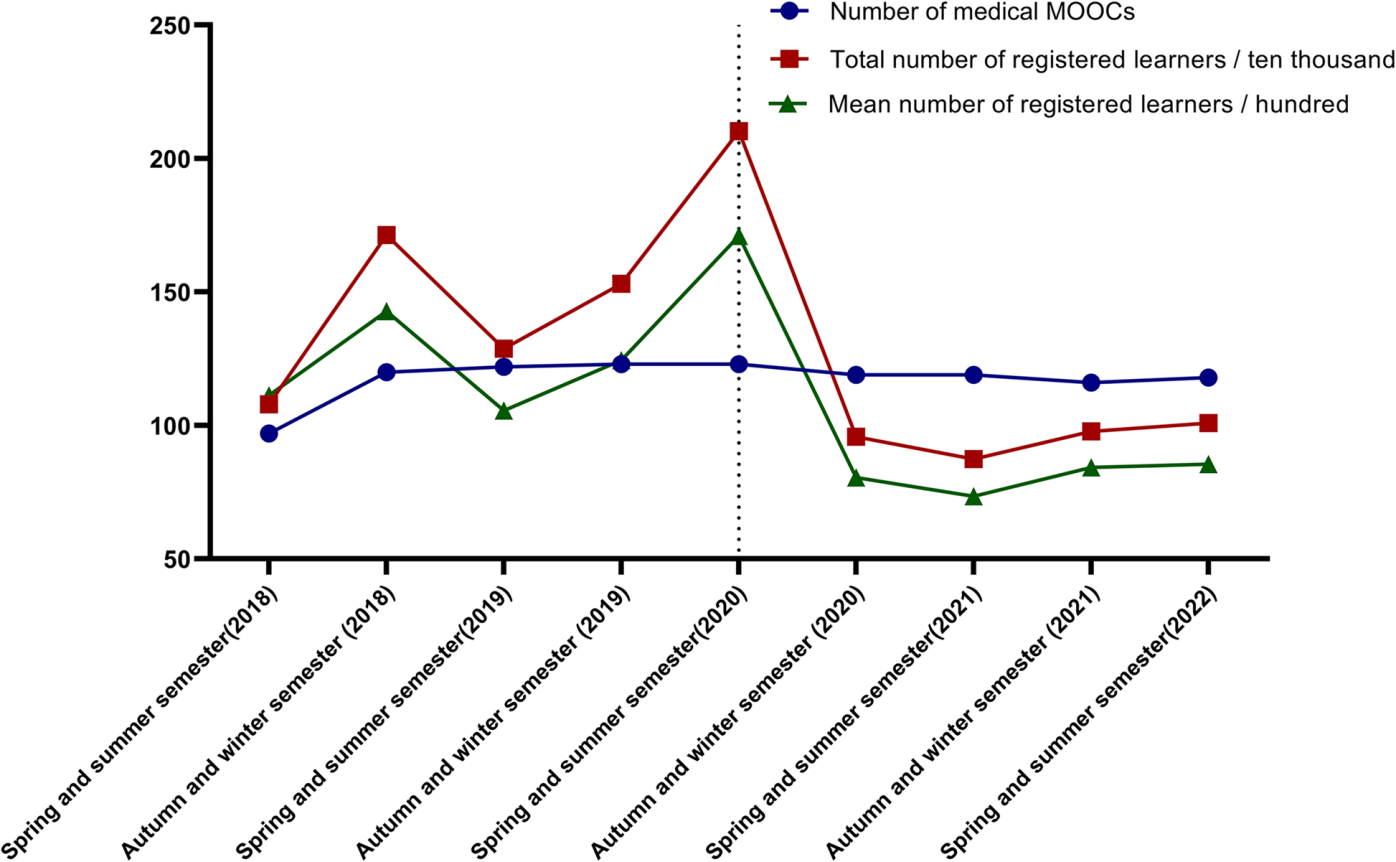
The numbers of registered learners of 141 national first-class medical MOOCs from 2018 to 2022

We obtained 141 national first-class medical MOOCs from the Smart Education of China Higher Education platform and analyzed the trend of the total and average number of registered learners for 9 semesters from the spring summer semester of 2018 to the spring summer semester of 2022. We found that the total and average numbers of registered learners of these national first-class medical MOOCs peaked in the spring–summer semester of 2020 (Fig. 4), suggesting that MOOCs played critical roles in higher education after college and university closures during the COVID-19 outbreak in the spring of 2020.

### Improved outcome indicators of medical MOOCs learning after 2020

The ‘Zhihuishu’ platform is the top 3 platform for medical MOOCs and recently included 674 medical MOOCs, among which national first-class MOOCs accounted for 6.7% (45/674). Moreover, we compared the differences of learning profiles and learning outcome indicators of medical MOOCs before and after 2020 based on 40 first-class medical MOOCs launched before 2020 in this platform. The median number of registered learners [3, 240 versus 2, 654], questions and answers [27, 005 versus 5, 116] and students taking the final examination [2, 782 versus 1, 995] of these MOOCs were significantly higher since 2020 compared to these before 2020, with *P* < 0.001 (Table 1).

**Table 1:** The learning profiles and learning outcomes of medical MOOCs from ‘zhihuishu” platform before and after COVID-19

Variables	Before 2020	Since 2020	*P*
Registered learners	2,645 (1,000–6,928)	3,240 (1,058–9,024)	**0.038**
Registered schools	22 (5–63)	19 (10–62)	0.600
Questions and answers	5,116 (1,105–15,823)	27,005 (5,822–101,117)	**0.000**
Students participating online discussions	796 (187–1,974)	722 (291–2,590)	**0.002**
Students taking the unit quiz	1,694 (644–6,240)	1,902 (727–6,918)	0.288
Students taking the final examination	1,995 (648–6,695)	2,782 (882–7,818)	**0.014**
Students passing the final examination	1,529 (477–5820)	1,885 (620–6,626)	0.183

All the variables were presented as median and inter-quartile range (IQR), and the differences between before 2020 and since 2020 were assessed with the Wilcoxon test

**Fig. 5:**
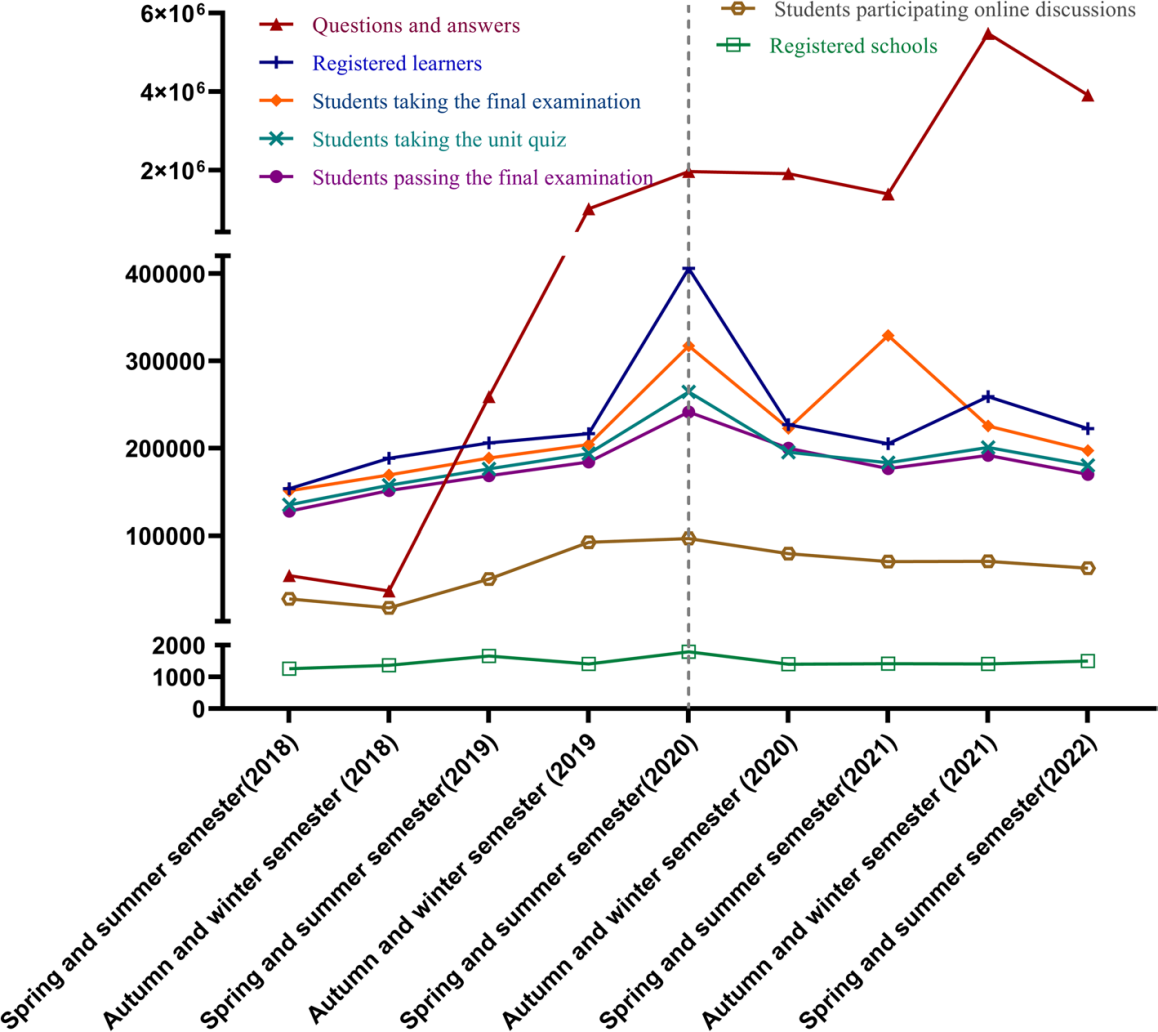
Dynamic changes in learning profiles and learning outcomes of medical MOOCs from 2018 to 2022. These records were obtained from the ‘Zhihuishu’ platform, and records of 40 national first-class medical MOOCs from this platform are presented in this figure

**Fig. 6:**
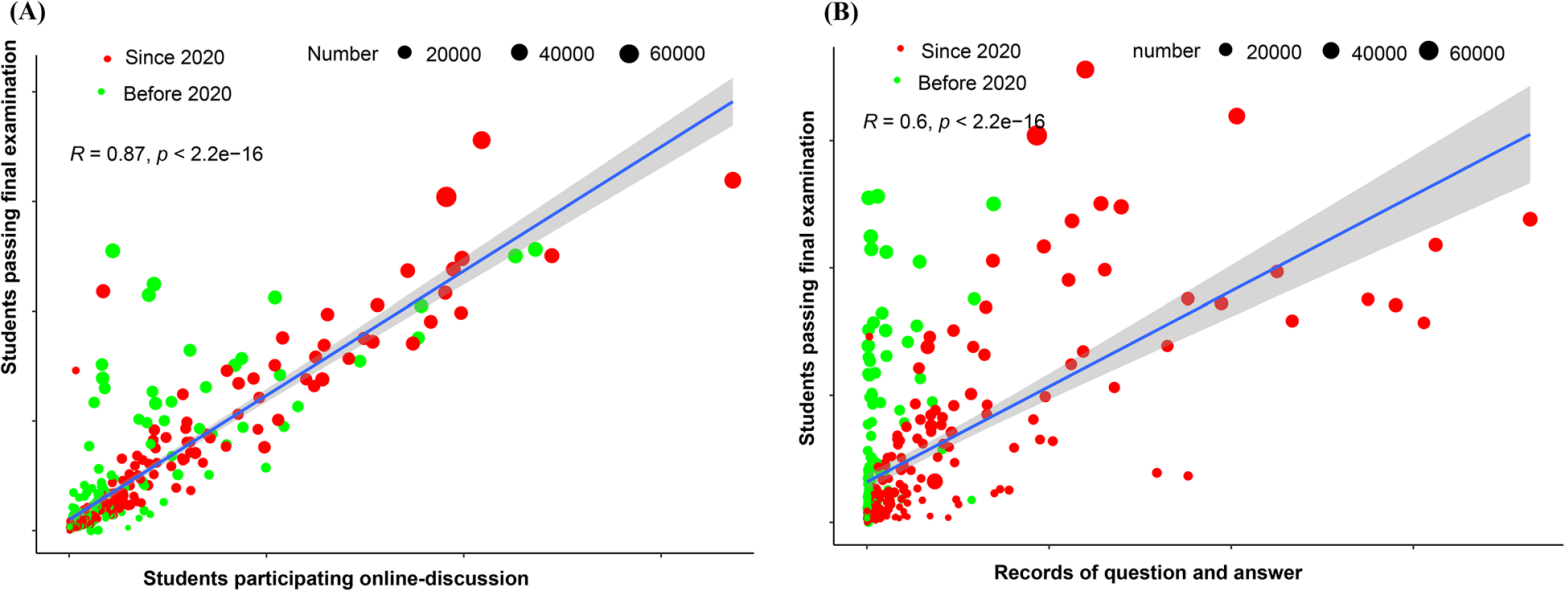
Correlation analyses of the records of online interaction and the number of passing examinations. **A** The correlation between the number of students participating online discussions and the number of passing final examinations. **B** The correlation between the records of questions/answers and the number of students passing final examinations

**Fig. 7:**
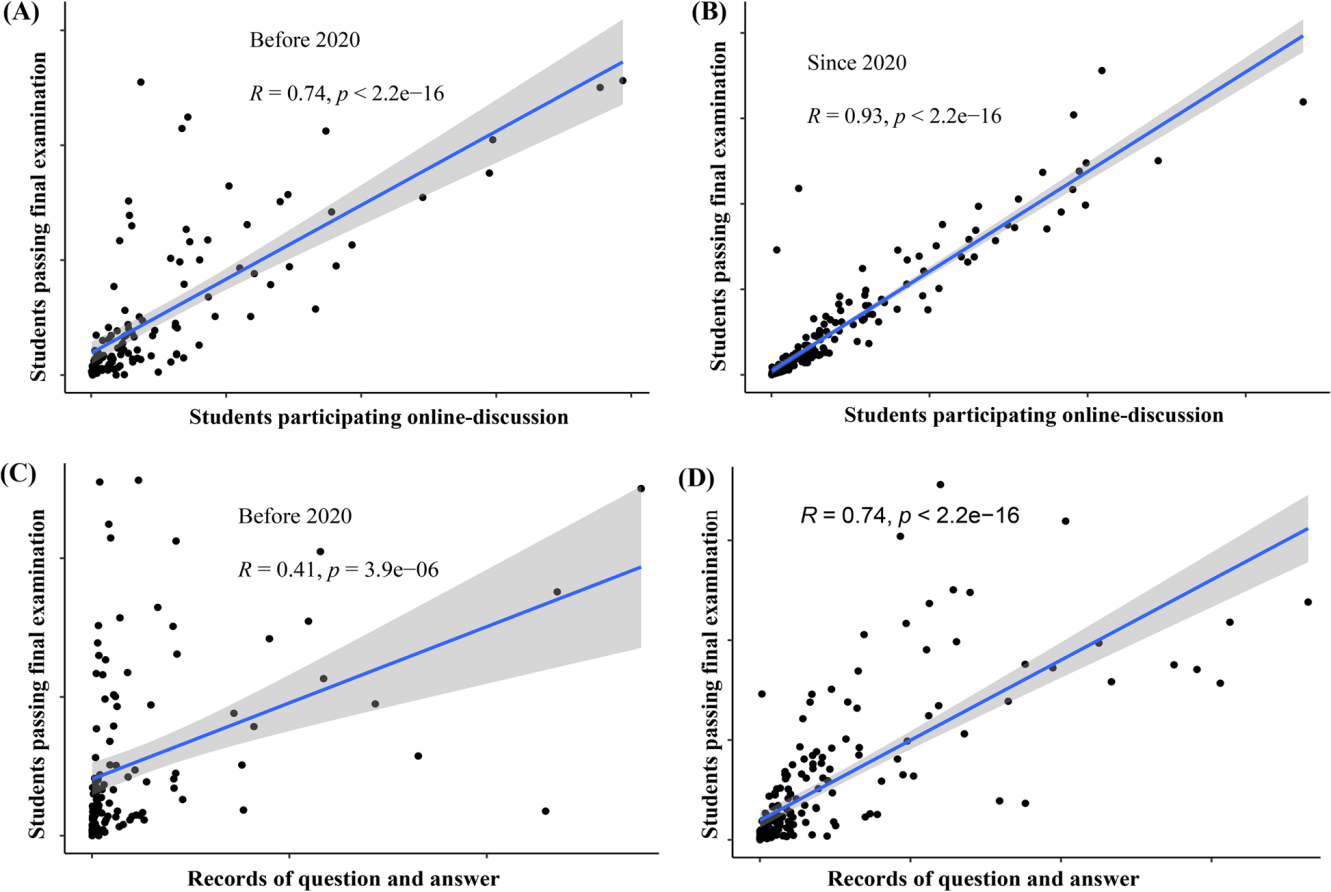
Correlation analyses of online interaction and the number of passing examinations before and after 2020. **A** The correlation between the number of students participating in online discussions and the number of passing final examinations before 2020. **B** The correlation between the number of students participating in online discussions and the number of passing final examinations since 2020. **C** The correlation between the records of questions/answers and the number of students passing final examinations before 2020. **D** The correlation between the records of questions/answers and the number of students passing final examinations since 2020

We further identified the dynamic changes in the number of registered learners, registered schools, students participating in online discussions, questions and answers, students taking the unit quiz, students taking the final examination, and students passing the final examination from the 2018 spring–summer semester to the 2022 spring–summer semester based on these 40 first-class medical MOOCs (Fig. 5). The data consistently showed that the number of registered learners, students taking the unit quiz, students taking the final examination and students passing examinations all peaked in the 2020 spring–summer semester. Questions and answers continued to rise since the fall-winter semester in 2018 and remained at a high level after 2020. Pearson's correlation analysis found that the records of questions and answers and the number of students participating in online discussions were both positively correlated with the number of students passing examinations (Fig. 6), and these correlations were stronger since 2020 (Fig. 7). Collectively, these data imply that MOOCs became the most important complementary approach to higher education during the COVID-19 pandemic and that online-classroom interaction on MOOC platforms effectively promoted MOOC learning effects in the pandemic scenario.

### The dramatic rise of medical MOOC-related publications since 2020

**Fig. 8:**
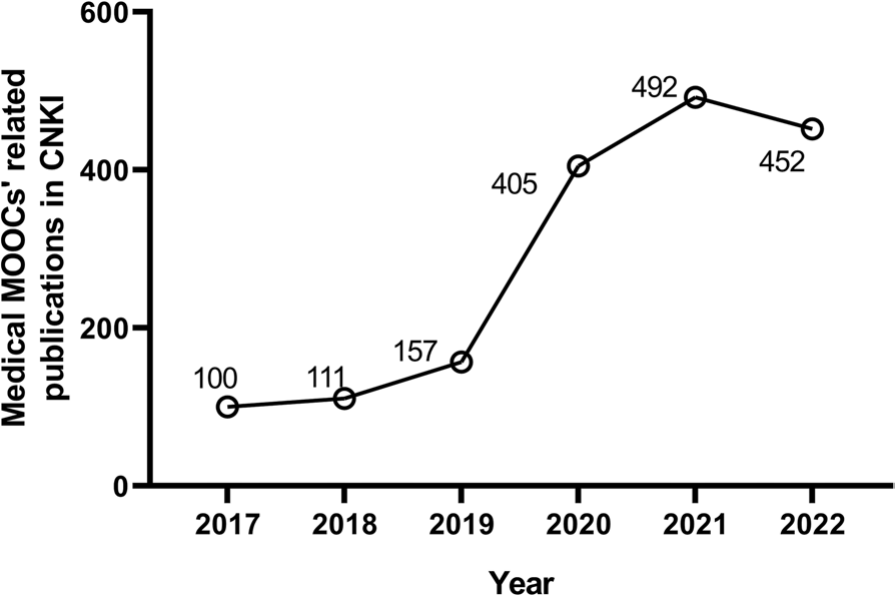
The rising trend of medical MOOC-related publications from 2017 to 2022. Medical MOOC-related publications were obtained from the China National Knowledge Infrastructure (CNKI)

CNKI is the largest academic resource knowledge service platform in China. Currently, CNKI covers more than 8,480 Chinese academic journals with 59.7 million academic studies and includes 5.6 million PhD/master’s dissertations and 3.6 million conference papers. We obtained medical MOOC-related publications from CNKI since January 2017. The results revealed that research in the field of medical MOOCs soared after 2020 and maintained a continuous upward trend (Fig. 8). Research on medical MOOCs has kept pace with the upsurge in MOOC applications in higher education since the COVID-19 crisis.

## Discussion

In the context of the ongoing COVID-19 pandemic, MOOCs have become reliable and valid digital sources to support higher education [25]. In this study, we explored the dynamic changes in medical MOOCs before and after the COVID-19 outbreak in China. The findings revealed that medical MOOCs have been launched rapidly since 2020, accounting for 70% of some China’s top online-education platforms. The number of participants of medical MOOC peaked during the spring–summer semester in 2020, and the learning performances were also remarkably improved since 2020. This was due to the suspension of traditional classroom teaching in China caused by COVID-19. The MOE issued a ‘suspension of classes without suspension of study’ in February 2020 [18]. Therefore, MOOCs were a useful approach for facilitating medical course dissemination at this stage. Given the popularity and acceptance of MOOC applications, the large-scale online asynchronous interaction between learners and instructors supports the teaching effect of medical MOOCs. MOOCs may help colleges and universities achieve a smooth transition from an online approach to classroom learning in the context of regular epidemic prevention and control of COVID-19. MOOCs have become a research focus, and the relevant literature about medical MOOCs has increased since 2020. This indicates that the educational value of medical MOOCs has been gradually emphasized in China.

The original pedagogical purpose of MOOCs as suggested by Downes highlighted social learning and educational equality through network interaction, especially expanded academic accessibility in global continuous education [26]. MOOCs can provide adequate and updated knowledge via free-time online access, and this novel teaching method appears to be effective and timely for professional education [27]. Similarly, MOOCs in the area of health and medicine have played a critical role in medical education, including continuing medical education programs, health care professional practices and campus-based medical higher education in recent decades [3, 28]. The consensus on medical MOOCs is that they open up avenues for continuing professional and interprofessional education for practitioners in the medical field and enhance health literacy among the public [29]. Although medical MOOCs have grown rapidly and are even considered the future of medical education, many current courses appear to be introductory in nature [30]. Therefore, some medical educators are suspicious of MOOCs’ strengths in medical school curricula among undergraduates, and they fear that medical MOOCs cannot satisfy the delivery of the dense medical information, skill and experience that is required in clinical practice [30]. Recently, MOOCs have been incorporated into medical higher education through blended learning, which facilitates the active learning model by combining online and in-person learning for students [31]. Overall, MOOCs represent an inspiring addition to medical higher education and practitioners’ continuing education training, but whether MOOCs can partially replace face-to-face classroom teaching remains uncertain.

The development of MOOCs in China has kept pace with the world trend. China’s MOOCs initially focused more on higher education, specifically campus education. Universities and colleges are the main force producing MOOCs, and educational institutions are committed to integrating MOOCs into the regular curriculum and creating a blended learning model [20]. In this study, we revealed the development and application of Chinese medical MOOCs, particularly the role of medical MOOCs in higher education before and after COVID-19 in 2020. Since China's first MOOC platform was launched in 2013, medical MOOCs have been continuously launched online, and their integration into school education is increasing. It is equally gratifying that large-scale, newly produced medical MOOCs were quickly uploaded as part of the rapid response to COVID-19. According to the results of this analysis, the scale of medical MOOC learners and online interactions peaked in 2020 during the initial spread of COVID-19, which indicated that the epidemic directly boosted the use of MOOCs in medical education.

The rapid expansion of digital learning in medical higher education after COVID-19 is not a chance occurrence. It benefits from the long-term construction of high-quality medical MOOCs and platforms in China [32, 33], which offered a solid basis for the transformation from traditional learning to MOOC learning during the enforced isolation stage in 2020. Moreover, MOOCs conform with the constructivist teaching mode, which highlights student-centered instruction [20]. This mode advocates that learning is not the passive transfer of knowledge from instructors to learners but an active process of seeking, absorbing and reconstructing knowledge with the help of instructors based on learners’ own knowledge background [21]. An early perspective in the *New England Journal of Medicine* in 2012 supported the flipped classroom mode in medical education because of the rapid growth of medical knowledge, which has caused even medical educators to suffer from information overload [31]. The highly diverse resources of MOOCs can facilitate autonomous learning and collaborative learning in students, which offers the opportunity to implement a new pedagogical approach. Additionally, the improved instructional design quality of medical MOOCs has helped to enhance students’ learning performance [34, 35]. For example, we evaluated medical MOOCs via participation, online interaction and learning performance on the ‘Zhihuishu’ platform. This Chinese MOOC platform mainly provides live interactive classes and video courses and designs online asynchronous question-and-answer boards that support learners in large-scale interactions. In terms of course examinations, medical MOOCs in the ‘Zhihuishu’ platform adopt multiple assessment methodologies and mandatory discussion forums, class performance, quizzes, unit tests and final examinations. This standard framework of medical MOOCs enhances voluntary and mandatory participation and facilitates the curriculum content of appraisal and evaluation. Before MOOCs integrate into or even replace formalized campus courses, valid indicators of the quality and application of MOOCs should be established. MOOC platforms need to release educational quality indicators, including teaching teams, instructional designs, learning goals, dynamic curriculum resources, discussion forums and learning outcomes. These learning variables are essential to determine the quality of MOOCs. Our analyses of the national first-class medical MOOCs on the ‘Zhihuishu’ platform showed that these MOOCs presented improved activation and utilization after 2020, especially during the COVID-19 pandemic. These might be mainly due to the favorable instructional design.

COVID-19 represented a teaching emergency in colleges and universities, and students were forced to shift from classroom learning to MOOCs in China during the initial spread of this virus [24]. Subsequently, the scale of MOOCs and registered participants in China ranked first worldwide after 2020. In fact, MOOCs have played an irreplaceable role in the emergency management of education and promoted higher education reform in China [36]. The number of medical MOOCs has consistently increased, and some have even suggested that MOOCs could replace traditional medical education in the near future, although this perspective is still widely controversial [25, 37]. The popularity of medical MOOCs is positively related to the perceived usefulness and perceived ease of use of MOOCs, which are important factors that motivate students’ independent learning [6, 38].

This study presented the rising trend and utilization of medical MOOCs before and after the COVID-19 outbreak in China, and demonstrated the irreplaceable role of medical MOOCs in teaching and learning under the policy of ‘stopping class without stopping learning’ during the pandemic. The data of this study were obtained from the Smart Education of China Higher Education platform. This was a nationally integrated MOOC platform established by China's MOE and comprehensively gathered high-quality MOOCs from other Chinese online-platforms, which ensured the representativeness and authenticity of data. Moreover, this study innovatively evaluated the different learning effects between medical MOOCs launched before and after 2020 based on 40 first-class medical MOOCs from ‘Zhihuishu’. Because ‘Zhihuishu’ provided complete set of teaching management resources for registration and performance management. Concurrently, the ‘Zhihuishu’ platform recently introduced medical microspecialties that gather top medical teams in China, including academics, medical experts and national scientists, and provide flexible and rigorous higher education in medicine via remote teaching [39]. Overall, the data analysis was persuasive based on ‘Zhihuishu’ platform. These results collectively supported the critical role and higher usage rate of MOOCs in medical education after COVID-19 pandemic.

Nevertheless, this study has several limitations. Firstly, the Smart Education of China Higher Education platform did not include all MOOCs from various online platforms. Therefore, the ratio of MOOCs launched after 2020 to those launched before 2020 was just 1.2 on Smart Education of China Higher Education platform, but the ratios of the ‘XuetangX’, ‘Xueying online’, ‘Zhihuishu’ and ‘iCourse’ platforms all exceeded 2.0 when all medical MOOCs were included. Secondly, this study was a retrospective study according to the data of the Smart Education of China Higher Education platform. There was a lack of prospective evidence to evaluate the different learning effects between MOOCs learning and traditional learning. Future studies are warranted to compare the different knowledge mastery between MOOCs learning and traditional face-to-face learning by prospective cohort design.

## Conclusions

In conclusion, high-quality medical MOOCs have been launched rapidly since the COVID-19 pandemic in China. The number of participants and online interactions in medical MOOCs peaked during the spring–summer semester in 2020. MOOCs are reliable and valid digital sources that facilitate medical higher education and play irreplaceable roles in emergency management.

## Supplementary Information

**Additional file 1.**

## Data Availability

The original data of the current study are publicly available on the Smart Education of China Higher Education platform (https://higher.smartedu.cn/) and Zhihuishu platform (https://www.zhihuishu.com/). MOOC URLs and links of 141 first-class national Medical MOOCs from the Smart Education of China Higher Education platform and 45 MOOCs with detailed learning outcomes from ‘Zhihuishu’ platform are presented in Supplementary Table 1.

## Abbreviations

MOOCs: Massive open online courses
COVID-19: Coronavirus disease 2019
MOE: Ministry of Education
CNKI: China National Knowledge Infrastructure
IQR: Interquartile range
